# The role of the peripheral and central adrenergic system in the construction of the subjective emotional experience of panic

**DOI:** 10.1007/s00213-024-06548-2

**Published:** 2024-02-16

**Authors:** Jette H. de Vos, Koen R. J. Schruers, Glen Debard, Bert Bonroy, David E. J. Linden, Nicole K. Leibold

**Affiliations:** 1 Department of Psychiatry and Neuropsychology, School for Mental Health and Neuroscience (MHeNs), Maastricht University, P.O. Box 616 (VIJV-SN2), 6200 MD Maastricht, The Netherlands; 2 Department of Health Psychology, University of Leuven, Leuven, Belgium; 3 Mondriaan Mental Health Center, Maastricht, The Netherlands; 4 Mobilab & Care, Thomas More Kempen, Geel, Belgium

**Keywords:** Emotions, Adrenergic beta-1 receptor, Panic, Carbon dioxide, Heart rate, Blood pressure

## Abstract

**Rationale:**

Although the study of emotions can look back to over 100 years of research, it is unclear which information the brain uses to construct the subjective experience of an emotion.

**Objective:**

In the current study, we assess the role of the peripheral and central adrenergic system in this respect.

**Methods:**

Healthy volunteers underwent a double inhalation of 35% CO_2_, which is a well-validated procedure to induce an intense emotion, namely panic. In a randomized, cross-over design, 34 participants received either a β_1_-blocker acting selectively in the peripheral nervous system (atenolol), a β_1_-blocker acting in the peripheral and central nervous system (metoprolol), or a placebo before the CO_2_ inhalation.

**Results:**

Heart rate and systolic blood pressure were reduced in both β-blocker conditions compared to placebo, showing effective inhibition of the adrenergic tone. Nevertheless, the subjective experience of the induced panic was the same in all conditions, as measured by self-reported fear, discomfort, and panic symptom ratings.

**Conclusions:**

These results indicate that information from the peripheral and central adrenergic system does not play a major role in the construction of the subjective emotion.

**Supplementary Information:**

The online version contains supplementary material available at 10.1007/s00213-024-06548-2.

## Introduction

Emotions allow us to communicate with our environment and help us learn which situations result in positive outcomes and hence should be approached, and which situations result in negative outcomes, and hence should be avoided. Emotions can be studied at different (psychological and physiological) levels, including subjective self-report (Kuppens et al. 2013), behavior, peripheral physiology (Scherer et al. 2001), and brain activation. Although the mechanisms of emotions have been studied for over a century, the question of how and based on which information the subjective experience of an emotion is constructed is still an unsolved puzzle (Moors 2009).

For a long time, opposing theories dominated reasoning on the role of peripheral physiological processes in the construction of subjective emotions. The James-Lange theory states that the perception of a situation results in bodily changes, and these bodily changes are equated to the emotion (e.g., we are afraid because we tremble) (James 1884). More recently, Damasio framed a similar theory, namely, the subjective feeling of an emotion results from perceiving what is going on in the body when you are experiencing an emotion (Damasio and Sutherland 1994). In Schachter’s two-factor theory, the peripheral physiological response is also necessary in the generation of emotions. According to this theory, the peripheral physiology is interpreted in the current context by cognitive processes and linked to a specific cause, leading to a specific subjective emotion (Schachter and Berkowitz 1967). For example, in the presence of a big dog fletching his teeth, one might attribute a raised heart rate to being afraid, while in the presence of a loved one, one might attribute the same raise in heart rate to being happy. The Cannon-Bard theory argues that the subjective experience of an emotion and the accompanying peripheral physiological response happen in parallel (Cannon 1927).

More recent theories have moved beyond these psycho-physiological dichotomies. The theory of constructed emotions of Barrett (2017) views an emotion as a whole brain-body phenomenon. More specifically, Barret argues that the brain predicts the cause of sensory events that detect irregularities in the extra personal world or internal milieu, and directs actions in order to maintain allostasis (Sterling 2012). During this process, previous experiences are taken into account. As reviewed by Moors (2009), there is a general trend of emotion theories to emphasize key roles for both subjective and physiological components as being part of an emotion, rather than equating one of those components to the emotion, which has not always been the case (LeDoux 2014). However, there is still no consensus on how the subjective emotion is constructed, and more specifically, which sources of information are taken as input in this construction.

An example of an intense emotion is panic, as it is felt during a panic attack (PA). PAs are characterized both by pronounced peripheral physiological and subjective components. Studies have reported an increased heart rate (Poonai et al. 2000; Richey et al. 2010; Schmidt et al. 1997), and strong increases in systolic (Leibold et al. 2013; Richey et al. 2010; Wetherell et al. 2006) and in diastolic blood pressure (Leibold et al. 2013; Richey et al. 2010). Moreover, sweating is one of the symptoms reported most often in relation to experiencing panic (Hoehn-Saric et al. 2004; Margraf et al. 1987; Meuret et al. 2011). With regard to subjective components, a feeling of overwhelming fear (Kessler et al. 2006) is one of the most prominent sensations. An inhalation of 35% CO_2_ induces short-lasting and self-limiting symptoms similar to the ones of a real life PA (Nardi et al. 2006; Schruers et al. 2004). A single vital capacity breath in patients with panic disorder (Esquivel et al. 2008; Nardi et al. 2006; Papp et al. 1993; Perna et al. 1995; Schmidt et al. 1997) and a double vital capacity breath in healthy people (Leibold et al. 2013; Schruers et al. 2011) are very well validated and widely used experimental models for panic (Leibold et al. 2015). Because of the available procedure to experimentally induce an intense emotion like panic, we will use this model in the current study.

The adrenergic system is involved in both the peripheral and central mechanisms present during an emotion. More specifically, an acute stressor quickly activates the locus coeruleus (LC), resulting in upregulation of noradrenaline levels (Rajkowski et al. 1997; Valentino and Van Bockstaele 2008). Additionally, the release of adrenaline from the adrenal medulla is triggered. Adrenaline and noradrenaline are important neurotransmitters in the peripheral, central, and autonomic nervous systems (PNS, CNS, and ANS O’Donnell et al. 2012; Wachter and Gilbert 2012)). For example, α- and β-adrenergic receptors are located on the heart and vascular smooth muscle, which explains the increased heart rate induced by confrontation with a stressor (Wachter and Gilbert 2012). In the CNS, noradrenergic neurons that originate in the LC project onto widespread areas across the cortex (O’Donnell et al. 2012), and are involved in changes in arousal, behavioral coping and alertness (Berridge 2008; Satpute et al. 2019).

Van den Hout and Griez (1984) have looked into the effects of blocking the adrenergic system on the subjective experience of panic. They found a reduction of CO_2_-induced panic symptoms in healthy subjects after administration of propranolol, a β-blocker that acts upon both the PNS and CNS. However, this does not allow disentangling the contributions of the peripheral and central adrenergic system to the construction of a subjective emotion. As a consequence, the unique contributions of the peripheral and central adrenergic system on the subjective experience of emotions remain unknown. The current study was designed to answer the question whether input from the peripheral adrenergic system is used in constructing the subjective experience of an emotion. More specifically, we will study the effects of peripheral β-receptor blockade, with a hydrophilic β-blocker that does not pass through the blood–brain barrier, on the subjective experience of experimentally induced panic in healthy individuals. Our primary hypothesis is that, because of the strong physiological and autonomic component of the panic response, peripheral β-receptor blockade (and thereby inhibition of these physiological responses) will attenuate the subjective experience of a CO_2_-induced PA, compared to a placebo. In order to study the unique contributions of the peripheral and central adrenergic systems to the subjective emotion, we would ideally also selectively inhibit the central noradrenergic activity. However, to the best of our knowledge, no pharmacological method exists to achieve this selective manipulation non-invasively in humans. Therefore, alternatively, a lipophilic β-blocker that passes through the blood–brain barrier and acts both in the peripheral and central nervous system will be used to explore the additive effect of inhibiting the central adrenergic system on top of the peripheral adrenergic system on the subjective experience of experimentally induced panic. Not only will this result in a better understanding of the interplay between psychology and physiology that constitutes an emotion, but also shed light into the best entry point for modulation of emotions (i.e., peripherally, or centrally) and thereby enhance treatments of emotion disorders.

## Materials and methods

### Pre-registration

Study proceedings, methods, and analysis strategies were preregistered on the Open Science Framework (https://osf.io/mz5k4).

### Participants

Fifty-four healthy volunteers (14 males) were recruited via advertisements at Maastricht University. Inclusion criteria were age between 18 and 55 years, weight between 55 and 100 kg, and a BMI ≥ 18.5 and ≤ 30.0. Exclusion criteria were current or past pulmonary or cardiovascular disease, current hypertension (systolic > 170 mmHg, diastolic > 100 mmHg), cerebral aneurysm, pregnancy or in lactation period, epilepsy, excessive smoking (> 15 cigarettes/day), current regular use of drugs and/or medication (except oral contraceptives), current or past psychiatric disorder (as assessed with the Mini International Neuropsychiatric Interview—Simplified (Sheehan et al. 1998)), and having a first-degree relative suffering from panic disorder. Additionally, in the first session, participants were screened for their sensitivity to the CO_2_ inhalation, as the degree to which panic symptoms are induced differs in healthy individuals (Battaglia et al. 2007). For the current study, only participants with a relatively high sensitivity to CO_2_ were included in order to be able to detect the differential effects of the drug interventions. Adequate sensitivity to a double 35% CO_2_ inhalation was defined as an increase of at least 25% on the Visual Analogue Scale-Fear (VAS-F) or Visual Analogue Scale-Discomfort (VAS-D) in addition to an increase of at least 1 on at least 4 symptoms listed in the Panic Symptom List (PSL; Leibold et al. 2013) compared to baseline. Thirty-four participants (11 males, mean age = 21.9 years, SD = 5.9) were included for the full study. Each subject gave written informed consent according to the principles of the Declaration of Helsinki. A financial compensation was paid for participation. The study was approved by the Medical Ethics Committee of the Maastricht University Medical Centre (study no. NL75335.068.20).

### Procedure

In a double-blind, randomized, placebo-controlled, within-subject design, subjects underwent four sessions. In the first session, the absence of exclusion criteria was confirmed, and a double 35% CO_2_ inhalation was performed to assess sensitivity to this procedure. If eligibility was confirmed, three additional sessions were scheduled, each one about 1 week apart, to assure that the drug of the previous session was washed out. Within each participant, the sessions started at the same time of the day to keep the effects of circadian rhythm on blood pressure constant (Douma and Gumz 2018).

The procedures of sessions 2–4 were almost identical. First, the participants were administered one oral tablet of atenolol (atenolol Sandoz, 100 mg), metoprolol (metoprolol tartrate Sandoz, 100 mg), or a placebo tablet. The order of conditions was counterbalanced. Next, there was a waiting period of 90 min. Dosage and waiting period were based on studies assessing pharmacological properties of atenolol (Baek et al. 2008; Fitzgerald et al. 1978) and metoprolol (Hemeryck et al. 2000). Each session was concluded with the CO_2_ inhalation. The CO_2_ procedure used here consists of a standardized double inhalation of a mixture of 35% CO_2_ (21% O_2_ balanced in N_2_, SOL Landgraaf, Nederland BV, the Netherlands), as described before (Leibold et al. 2013). Briefly, subjects exhaled as much as possible, inhaled as much as possible, exhaled and inhaled again, and held their breath for 4 s.

### Self-report measures

Immediately before and after the inhalation, self-report measures were taken (in line with previous CO_2_ studies: e.g., Leibold and colleagues (Leibold et al. 2013)). After the inhalation, participants were instructed to base their rating on the most intense moment of the inhalation. First, participants rated their subjective feelings of fear and discomfort by means of a Visual Analogue Scale (VAS-F and VAS-D, respectively). The scales consist of a horizontal line that ranges from 0 (not at all) to 100 (the worst imaginable).

Then, participants evaluated panic symptoms using the Panic Symptom List (PSL), which lists the 13 symptoms characterizing a PA according to the DSM-V criteria American Psychiatric Association (2013) such as palpitations, shortness of breath, feeling of choking, dizziness, and fear of losing control or of dying. The presence of each item is rated on a 5-point Likert scale, from 0 (absent) to 4 (very intense).

At the end of the last session, a short questionnaire was administered, to explore the awareness of the participants towards the aims and used conditions of the experiment.

### Physiological measures

Blood pressure (BP) and heart rate (HR) were measured using an Omron HBP 1120 professional BP monitor (Omron Healthcare Co., Ltd. Kyoto, Japan). Measurements were performed right before and after the CO_2_ inhalation. Additional measurements were performed at the start of the session, 30, 60, and 90 min after drug intake.

Additionally, we used the Empatica E4 wristband (Garbarino et al. 2014; McCarthy et al. 2016; Ollander et al. 2016). The Empatica E4 measures blood volume pulse (BVP) via a photoplethysmography (PPG) with a sample rate of 64 Hz, from which HR can be derived. Participants wore the Empatica E4 wristband during the whole sessions. Tags were applied in the recording to be able to segment the data for later analysis.

### Statistical analysis

R version 4.0.3 (R Development Core Team 2010) was used to organize the data in preparation for subsequent analyses; all statistical analyses were done using SPSS Version 27 (IBM Corp 2020).

First, the effect of the drug conditions on the physiological measurements was assessed, to confirm the effectiveness of the drugs to induce HR and BP reductions, compared to the placebo condition. Three random intercept/random slope multilevel models were tested, with an unstructured variance–covariance matrix, to account for the repeated measures nature of the study, with diastolic BP, systolic BP, and HR as dependent variables. Condition (atenolol, metoprolol, and placebo) and timepoint of the measurement (0, 30, 60, and 90 min after drug intake) were taken as fixed factors. Participants were taken as a random factor. In all multilevel models reported in this study, the following model was tested (subscript *i* refers to the participant and subscript *j* to the measurement):

$${\mathrm{Outcome}}_{ij}=\beta_{0i}+\beta_{1i}\ast{\mathrm{Timepoint}}_{ij}+\beta_{2i}\ast{\mathrm{Condition}}_{ij}+\beta_{3i}\ast{\mathrm{Timepoint}}_{ij}\ast{\mathrm{Condition}}_{ij}+R_{ij}$$

Follow-up paired samples *t*-tests were performed when overall tests were significant. The measurements taken at the end of the waiting period (90 min after drug intake) were of particular interest to confirm the effectiveness of the manipulation.

In parallel, paired samples *t*-tests were performed to confirm the effectiveness of the drugs to reduce HR during the inhalation, as measured continuously with the Empatica E4 wristband. To calculate HR, first, the BVP signal was cleaned using a second-order bandpass filter with cutoff frequencies of 22.5 and 200 bpm. This was followed by a median filter (filter length 35) and a mean filter (filter length 35) to remove noise. The interbeat intervals (IBI) were measured as the time between the subsequent signal peaks. Invalid IBIs, caused by missing data, were removed. From these IBIs, HR was subsequently calculated as the reciprocal of the IBI. Second, mean HRs were computed per condition (atenolol, metoprolol, and placebo), averaged from 5 to 30 s after the start of the inhalation (the first 5 s of the inhalation were excluded to remove noise induced by pressing the button on the wristband, which was pressed to add a tag in the data). A random intercept/random slope multilevel model with an unstructured variance–covariance matrix was performed to assess whether the drugs induced differences in the increase in HR during the inhalation. Condition (atenolol, metoprolol, and placebo) and timepoint of the measurement (baseline, during the inhalation) were taken as fixed factors. Participants were taken as a random factor. The baseline was defined as the mean HR over a period of 2 min, sampled at the end of the waiting period, when participants were sitting and listening to an emotional neutral podcast.

Second, paired samples *t*-tests were performed to confirm the effectiveness of the inhalation to induce changes in subjective ratings. Change scores for fear, discomfort, and panic symptom ratings were computed per session, by subtracting ratings prior to the inhalation from the ratings given after the inhalation. Additionally, the influence of the different conditions on this subjective experience of the inhalation procedure was examined with random intercept/random slope multilevel models with an unstructured variance–covariance structure. Three multilevel models were tested, one for each of the subjective ratings, with the condition (atenolol, metoprolol, and placebo) and timepoint of the measurement (pre- and post-inhalation) included as fixed factors. Participants were taken as a random factor. Follow-up *t*-tests were performed, using Holm’s method to control for family-wise errors (Holm 1979), when overall tests were significant, defined as *p* < 0.05.

Pearson correlation coefficients were computed between physiological and subjective outcome measures to check for within-session correlations. Holm’s method to control for family-wise errors (Holm 1979) was applied.

## Results

### Manipulation check of drug conditions

**Fig. 1:**
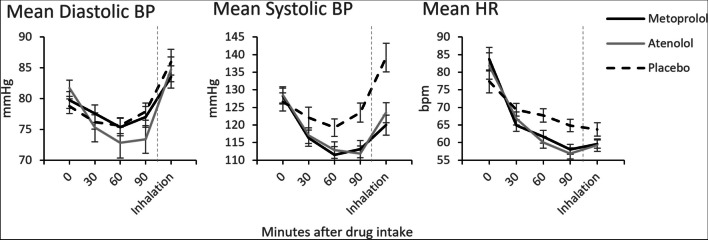
Mean values of physiological parameters for the different conditions, over the course of the waiting period, starting at drug intake. Error bars represent the standard error of the mean. BP blood pressure, HR heart rate. For distribution of the data, see Fig. S1

In order to test the effectiveness of the drug manipulation in inhibiting the noradrenergic system, and thereby reducing BP and HR, we analyzed HR and BP over the course of the experiment, by means of multilevel models. For HR and diastolic and systolic BP, there was a significant condition × time interaction (*F* = 2.51, *p* = 0.022; *F* = 3.89, *p* = 0.001; and *F* = 2.45, *p* = 0.047, respectively, illustrated in Fig. 1). Follow-up *t*-tests confirmed the effectiveness of the β-blockers in reducing the physiological parameters at the end of the waiting period (90 min after drug intake). As documented in Table 1, diastolic BP is significantly lower in the atenolol condition than in the placebo condition. Diastolic BP in the metoprolol condition did not differ from the placebo condition. Systolic BP and HR are significantly lower in both the atenolol and metoprolol condition compared to the placebo condition. There are no significant differences between the atenolol and metoprolol condition.

**Table 1:** Paired samples *t*-tests of physiological parameters

Condition	Placebo	Atenolol	Metoprolol
Placebo DIAS SYS HR		***t*** = 3.21* *t* = 4.78*** *t* = 3.30*	*t* = .407 *t* = 7.40*** *t* = 4.50***
Atenolol DIAS SYS HR			*t* = − 1.44 *t* = − .465 *t* = − .943
Metoprolol DIAS SYS HR			

Paired samples *t*-tests of physiological parameters (*DIAS* diastolic blood pressure, *SYS* systolic blood pressure, *HR* heart rate) measured at the end of the waiting period, 90 min after drug intake, between the different drug conditions. For all *t*-tests, the *df* = 33. *p < .05, **p < .01, ***p < .001

Additionally, the diastolic BP measured right after the inhalation did not differ between the conditions (atenolol vs. placebo, *t*(33) = 0.54, *p* = 0.592; metoprolol vs. placebo, *t*(33) = 0.94, *p* = 0.355; atenolol vs. metoprolol, *t*(33) = 0.598, *p* = 0.554). Systolic BP was lower in the atenolol and metoprolol sessions compared to the placebo session (*t*(33) = 5.08, *p* < 0.001; *t*(33) = 5.61, *p* < 0.001, respectively). The systolic BP did not differ between the atenolol and metoprolol session (*t*(33) = 1.18, *p* = 0.245). Similarly, HR was lower in the atenolol and metoprolol sessions compared to the placebo session (*t*(33) = 2.60, *p* = 0.014; *t*(33) = 2.70, *p* = 0.011, respectively). HR was similar between the atenolol and metoprolol session (*t*(33) =  − 0.16, *p* = 0.873).

### Effects of drug conditions on physiological measures during experimentally induced panic

**Fig. 2:**
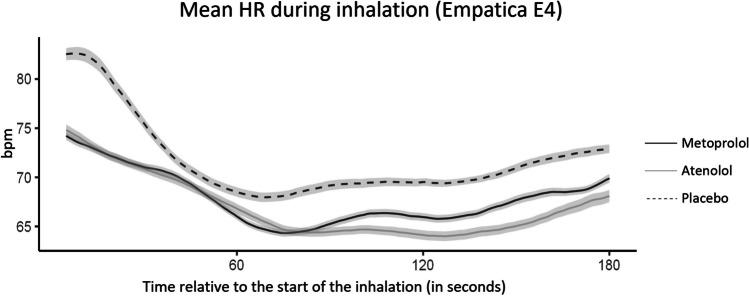
Mean heart rate time course during the CO_2_ inhalation, as measured with the Empatica E4 wristband. Error bars represent the standard error of the mean. HR heart rate

HR was measured continuously during the inhalation with the Empatica E4 wristband (Fig. 2). When looking at HR data during the inhalation, again, the average HR in the atenolol and metoprolol sessions was lower compared to the placebo session (*t*(34) = 4.23, *p* < 0.001; *t*(34) = 3.87, *p* < 0.001, respectively). HR did not differ between the atenolol and metoprolol session (*t*(34) = 0.12, *p* = 0.909). The relative increase in HR during the inhalation, compared to baseline, did not differ between the conditions, as shown by a non-significant condition × time interaction term in the multilevel model (*F* = 1.34, *p* = 0.266).

### Effects of drug conditions on subjective experience of experimentally induced panic

**Fig. 3:**
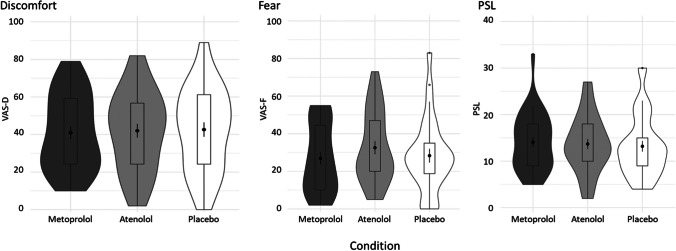
Violin plots representing the changes in subjective ratings (VAS-D, VAS-F, and PSL) from prior to to right after the inhalation procedure for the different conditions

Next, we tested the effects of the inhalation and drug manipulation on the subjective experience of the experimentally induced panic. As shown in Fig. 3, the inhalation procedure was effective in causing an increase in all three outcome measures (VAS-D, *mean increase* = 41.15, *t*(34) = 13.33, *p* < 0.001; VAS-F, *mean increase* = 23.07, *t*(34) = 6.80, *p* < 0.001; PSL, *mean increase* = 13.05, *t*(33) = 13.47, *p* < 0.001). However, for all three outcome measures, there was no significant condition × time interaction (VAS-D, *F* = 0.077, *p* = 0.926; VAS-F, *F* = 0.234, *p* = 0.791; PSL, *F* = 0.340, *p* = 0.712). Additionally, there was no main effect of condition (VAS-D, *F* = 0.721, *p* = 0.488; VAS-F, *F* = 0.376, *p* = 0.688; PSL, *F* = 1.405, *p* = 0.249). In other words, there is no difference in the subjective experience of the CO_2_ inhalation between the two β-blockers compared with placebo. Moreover, there is no difference in the subjective experience when comparing the two β-blockers.

Induced changes in physiological and subjective parameters were not correlated (see Supplementary Results Tables S1).

## Discussion

In the current study, we address the unique contributions of the central and peripheral adrenergic system to the subjective experience of an emotion. With a hydrophilic and a lipophilic β-blocker, we inhibited noradrenergic activity in either the periphery or in both the peripheral and central nervous system (PNS and CNS, respectively), before inducing short-lasting panic with a 35% CO_2_ inhalation. The β-blockers successfully reduced HR and systolic BP. Furthermore, participants reported increased levels of fear, discomfort, and panic symptoms in response to the CO_2_ inhalation, in line with the experiences during a naturally occurring PA and as defined in the DSM criteria for a PA (Griez et al. 2007; Leibold et al. 2016; Schruers et al. 2004). However, the subjective experience of the inhalation was the same in all conditions. These results suggest that information from the peripheral and central adrenergic system does not contribute to the construction of the subjective emotion.

In line with our results, several studies report a dissociation between peripheral physiological and subjective correlates of panic. If peripheral physiological information would be used in the construction of a subjective emotion, one could expect to observe a reduction in peripheral physiological parameters when the subjective emotion is reduced and vice versa. In line with our results, several other studies did not find an association between peripheral physiological and subjective correlates of an emotion. For example, Salvadore et al. (2020) found a reduced subjective response to the 35% CO_2_ inhalation after administration of a selective orexin-1 receptor antagonist. However, the measured cardiovascular parameters were not affected. In line with this, Leibold et al. (2013) found a dose-dependent increase in subjective outcomes in the absence of a dose-dependent increase in HR. In a meta-analysis summarizing the effects of the CO_2_ inhalation, an increase in self-reported subjective distress due to the CO_2_ inhalation was consistently found. On the other hand, results regarding cardiovascular parameters were more heterogenous, with no significant overall effect (Liu et al. 2019).

The second aim of this study was to explore the additive effect of inhibiting the adrenergic system in the central nervous system, on top of selectively inhibiting the adrenergic system in the peripheral nervous system. As mentioned above, the subjective experience of the inhalation was the same in all conditions. Because we found no difference between peripheral β-blockade alone and both peripheral and central β-blockade, the results suggest that neither the peripheral nor central adrenergic system has a sole contribution to the construction of the subjective experience of the panic response.

The study design of Van den Hout and Griez (1984) looked at the effects of the β-blocker propranolol (acting both in the PNS and CNS) on subjective panic responses. Contrary to the current results, participants reported less intense panic symptoms when the adrenergic system was inhibited. However, a small sample size was used and no comparison was made with a β-blocker selectively acting in the PNS. The discrepancy in the results could be explained by the different types of β-blockers we used in the current study. The β-adrenoreceptor sub-class comprises β_1_- and β_2_-receptors (Lands et al. 1967). Propranolol is a β-blocker that effects both receptor types. Atenolol and metoprolol on the other hand only influence the β_1_-receptor type. A deliberate choice was made to use β_1_-selective β-blockers in the current study. First, there is no suitable readily available non-selective hydrophilic β-blocker. For example, nadolol is not registered in the Netherlands, and sotalol possesses additional non β-blocking effects which could confound the results (Singh and Nademanee 1987). Therefore, we selected atenolol in order to be able to examine the effects of inhibiting the peripheral adrenergic system on the subjective emotion. In order to keep the two β-blocker conditions as similar as possible, apart from the lipophilic and hydrophilic properties, we also used a β_1_-selective lipophilic β-blocker, metoprolol.

The second reason for using β_1_-selective β-blockers was avoiding the blocking of β_2_-receptors located on the bronchi (Cruickshank 1980). In light of the hypothesis of apnea-induced anxiety (Feinstein et al. 2022), which highlights the involvement of respiratory parameters in panic, the difference between β_1_-selective and non-selective β-blockers can be relevant. This hypothesis states that the amygdala induces episodes of apnea that cause elevated levels of CO_2_, which result in compensatory changes in respiration and anxiety or panic. In that framework, inhibition of respiratory parameters by a non-selective β-blocker (Van den Hout and Griez 1984) might reduce subjective panic, while selective inhibition of β_1_-receptors might not. A way to test this would be by directly comparing the effects of selective and non-selective β-blockers on the subjective experience of panic. Importantly, respiratory parameters should also be measured to detect a potential association with the subjective emotion.

While the β-blockers successfully reduced the absolute HR and BP values at the time of the inhalation, the relative increase in HR, when comparing HR during the inhalation with baseline, was not affected. Therefore, we cannot rule out that relative changes in HR could be used as input for the construction of the subjective experience of panic. There might be another (neurotransmitter) system involved in this stress-induced increase in HR. Alternatively, the β_1_-blockade might not be strong enough to inhibit stress-related adrenergic activity, despite successful inhibition of tonic adrenergic activity. Although it is still possible that this transient increase in HR triggered the emotional experience, a prominent effect of β_1_ receptors in the brain is unlikely because there was no difference in the panic response between atenolol and metoprolol.

A limitation of the current study is that we are unable to compute HR variability. Although HR did not seem to align with the subjective responses in the study of Leibold et al. (2013), HR variation showed a clear dose-dependent increase similar to the subjective response. Yet, HR in the current study was measured with the Empatica E4 wristband. This wristband is extremely suitable for use during the CO_2_ inhalation: when worn correctly, it is relatively unsensitive to movement and it does not require a complex lab setup which could confound the panic experience. However, the sampling rate of our HR measurement device is not suitable for computing HR variability (Ziemssen et al. 2008).

In conclusion, the current study tested the effect of inhibiting the peripheral and central adrenergic systems on the subjective experience of an emotion. Successful inhibition of adrenergic tone did not affect the subjective experience of panic, or its associated autonomic response, transiently increased heart rate. Although we cannot rule out that peripheral physiological components have a role in constructing the subjective experience of panic (Damasio and Sutherland 1994; James 1884), these findings make a major role of β_1_- receptors, particularly those in the CNS, rather unlikely. It thus seems that we need to look beyond the adrenergic system both for understanding the construction of a subjective experience of an emotion and potentially new treatments of panic disorder.

### Supplementary Information

Below is the link to the electronic supplementary material.

Supplementary file1 (DOCX 146 KB)
