# Supplementary material for: The role of the peripheral and central adrenergic system in the construction of the subjective emotional experience of panic
Source: Psychopharmacology (Berl). 2024 Feb 16;241(3):627–35. doi: 10.1007/s00213-024-06548-2 (PMC10884065; doi:10.1007/s00213-024-06548-2)
Supplement: Supplementary file 1 — Supplementary file1 (DOCX 146 KB) [file 213_2024_6548_MOESM1_ESM.docx]

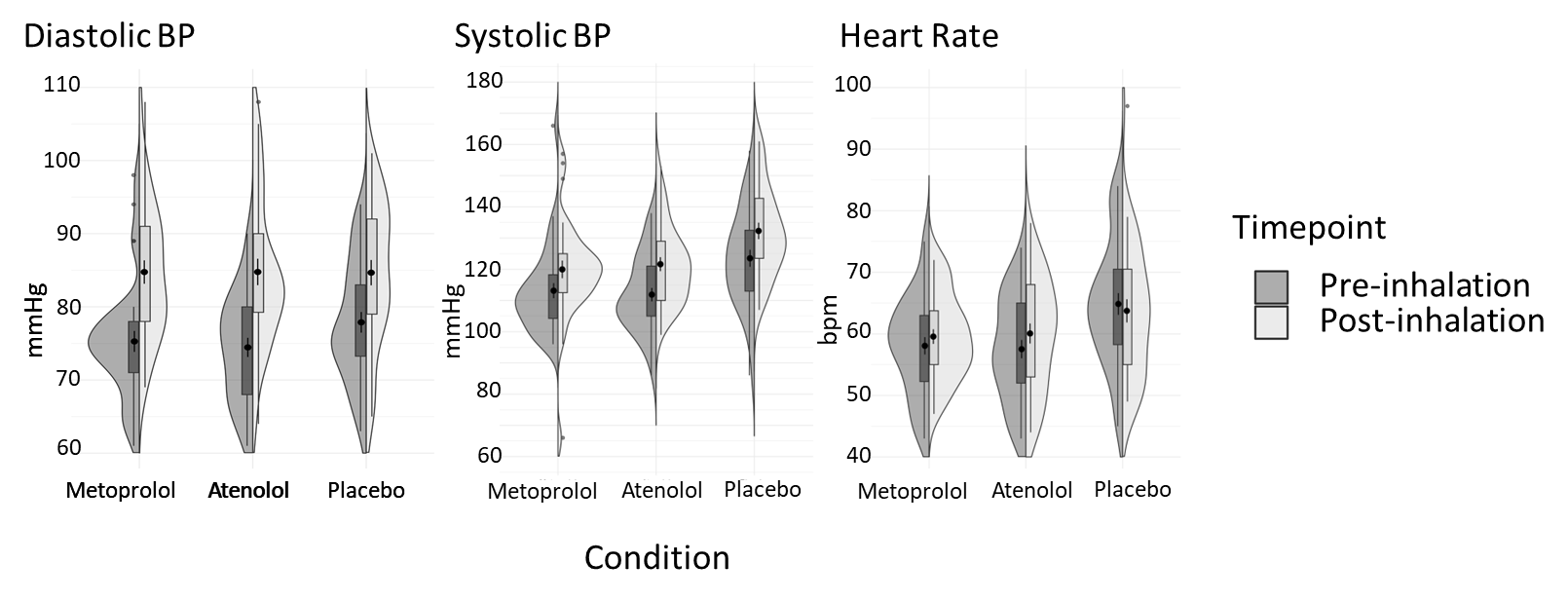


**Fig. S1.** Violin plots representing the physiological parameters (diastolic and systolic blood pressure, heart rate) measured before the inhalation (i.e, 90 minutes after drug intake) and immediately after the inhalation for the different conditions.

**Table S1. Within-session correlations between physiological and subjective outcome variables**

|  | **Atenolol** | | |  | **Metoprolol** | | |  | **Placebo** | | |
| --- | --- | --- | --- | --- | --- | --- | --- | --- | --- | --- | --- |
|  | SYS_  change | DIAS_  change | HR_  change |  | SYS_  change | DIAS_  change | HR_  change |  | SYS_  change | DIAS_  change | HR_  change |
| VAS-D_  pre | -.227 (.197) | -.009 (.961) | .020 (.912) |  | .117 (.511) | -.014 (.936) | -.098 (.589) |  | -.022 (.904) | .249 (.155) | -.239 (.180) |
| VAS-D_  post | -.173 (.328) | -.297 (.088) | .159 (.376) |  | .096 (.589) | -.006 (.973) | -.015 (.833) |  | .267 (.127) | .189 (.284) | .168 (.349) |
| VAS-D_  change | -.099 (.576) | -.286 (.101) | .163 (.366) |  | .021 (.908) | -.013 (.942) | .034 (.853) |  | .237 (.177) | .038 (.830) | .294 (.097) |
| VAS-F_  pre | -.057 (.750) | .175 (.322) | -.083 (.647) |  | -.093 (.599) | -.140 (.429) | -.126 (.486) |  | .082 (.647) | .087 (.624) | .127 (.481) |
| VAS-F_  post | -.014 (.936) | .053 (.765) | .295 (.095) |  | .170 (.337) | .143 (.421) | .067 (.711) |  | .283 (.105) | .330 (.056) | .137 (.446) |
| VAS-F_  change | -.006 (.972) | -.076 (.670) | .305 (.084) |  | .211 (.232) | .243 (.166) | .169 (.348) |  | .220 (.212) | .245 (.162) | .025 (.888) |
| PSL_  pre | -.263 (.132) | -.320 (.065) | -.028 (.878) |  | .130 (.465) | -.054 (.761) | .100 (.580) |  | -.032 (.858) | .125 (.480) | -.096 (.593) |
| PSL_  post | -.196 (.268) | -.047 (.792) | .028 (.878) |  | .073 (.686) | .051 (.780) | -.127 (.478) |  | -.052 (.771) | .249 (.156) | .027 (.882) |
| PSL_  change | -.284 (.110) | -.062 (.730) | .023 (.902) |  | -.075 (.680) | .001 (.996) | -.225 (.216) |  | -.088 (.620) | .207 (.239) | .049 (.786) |

Within-session Pearson correlations (Sign. (2-tailed)) between physiological and subjective outcome variables. No correlations were significant after Holm’s method to control for family wise errors. VAS-D = Visual Analogue Scale Discomfort; VAS-F = Visual Analogue Scale Fear; PSL = Panic Symptom List; SYS = Systolic Blood Pressure; DIAS = Diastolic Blood Pressure; HR = Heart Rate, continuously measured with the Empatica E4.
